# Reassessing Sexual Orientation Change Efforts: A Call for Evidence-Based and Culturally Sensitive Discourse

**DOI:** 10.7759/cureus.84636

**Published:** 2025-05-22

**Authors:** Jaafar O Ahmed, Karwan Kakamad

**Affiliations:** 1 Psychology, Soran University, Soran, IRQ; 2 Scientific Research, Kscien Organization, Sulaimanyah, IRQ

**Keywords:** cultural and religious sensitivity, evidence, same-sex attraction, sexual orientation, soce

## Abstract

The need for evidence-based, culturally aware, and morally sound discourse is emphasized in this paper's critical re-examination of Sexual Orientation Change Efforts (SOCE). Drawing on recent empirical data from a sample of 72 men who self-identified as religious, the paper highlights subtle and modest findings that suggest possible changes in sexual attraction and behavior without making broad claims about efficacy. There are currently few longitudinal and demographically diverse studies to support clinical or policy consensus, and the field of SOCE research is still fragmented. This paper makes the case that prevalent institutional positions, which frequently reject SOCE categorically, are occasionally supported by socio-political or moral arguments rather than thorough empirical data. Additionally, it emphasizes how crucial it is to take into account religious and cultural contexts in therapeutic settings, as some people may voluntarily seek change-oriented support due to internal value conflicts and individual autonomy. To distinguish between coercive or harmful practices and ethically carried out, client-directed interventions, the discussion advocates for a more inclusive, scientifically rigorous approach to SOCE research. In the end, the paper promotes a multifaceted interpretation of SOCE that incorporates therapeutic autonomy, cultural sensitivity, and empirical validity.

## Editorial

Sexual Orientation Change Efforts (SOCE) have long been a contentious topic in the discourse surrounding mental health, psychiatry, public health, and human rights. Sullins (2024) discusses this subject in his most recent paper, "What Sexual Orientation Change Efforts Change: Evidence from a United States Sample of 72 Exposed Men." He came to the conclusion that homosexual study participants' changes in sexual orientation primarily led to an increase in heterosexual orientation and a decrease in same-sex attraction and behavior [1]. Sullins' study separated the specific elements of reported change - behavior, attention, and romantic ideation - in an attempt to go beyond broad claims of efficacy. It calls attention to this issue, where prevailing institutional guidelines have frequently been made without sufficient empirical consensus, despite the results being modest and demographically limited [2,3]. The study does not refute worries about possible harm or support SOCE as a widely used, successful intervention. Instead, it poses an important and crucial question: rather than being founded on political or moral consensus, clinical and policy guidelines about SOCE should be informed by growing scientific evidence [1].

The literature on SOCE is still dispersed at the moment. Others draw attention to possible psychological risks, especially in coercive or aversive contexts [3], even though Sullins' study and others found apparent positive outcomes among some populations [4,5]. Positive outcomes of SOCE include changes from a predominantly homosexual orientation to a predominantly heterosexual orientation, which consists of a reduction in homosexual attraction and behavior and an increase in heterosexual attraction and behavior [1,4,5]. On the SCOE, there is some evidence; therefore, there is a knowledge gap regarding the entire spectrum of SOCE-related effects due to the dearth of robust, varied, and long-term studies. This paper aims to urge more effort and work to provide sufficient evidence based on new and unique empirical research.

Cultural and religious contexts: a neglected dimension

One of Sullins' research's most notable features is identifying the religious and cultural factors contributing to many people's detection of SOCE. Most of the study's participants were religious men; more than 80% of them said they regularly attended religious services weekly, which is much higher than the national average for both the general and lesbian, gay, bisexual, and transgender (LGBT) populations. Similarly, 91% of the participants were white [1]. This demographic domain significantly impacts clinical practice. Many people's experiences with sexual identity and orientation are not isolated; instead, they are closely linked to their public, familial, and spiritual frameworks. If these intersections are ignored, clients whose psychological distress results from internal value conflict rather than external repression may go unnoticed [2]. Resolving these conflicts in favor of religious analogy may be a reasonable therapeutic goal for those individuals, provided the efforts are voluntary, morally righteous, and non-aversive. One of the fundamental bioethical principles is respect for autonomy, which calls for acknowledging a person's right to express their therapeutic objectives, even if those objectives deviate from critical cultural norms. It may be considered culturally insensitive or even logically unfair to deny someone access to therapeutic support for their distress caused by unwanted same-sex attraction because of preconceived notions about what constitutes an acceptable identity [3].

Scientific truth and the ethics of evidence

Numerous psychological associations, such as the American Psychological Association (APA), have categorically banned SOCE in recent years, often citing the efforts as fundamentally harmful and lacking scientific validity [2]. However, as Sullins and others have argued [4,5], the evidence supporting these broad prohibitions is still incomplete; firstly, banning SOCE violates the autonomy principle of bioethics [1,2,5]; secondly, numerous published papers in the past have suggested that homosexual orientations can change [2,4,5]; and lastly, due to these prohibitions, there is a lack of recent studies addressing the harm caused by SOCE [1,5]. Thus, the important ramifications of the Sullins study also affect the ethical and philosophical frameworks that direct research, policy, and professional practices [1].

While safeguarding vulnerable individuals from harmful or non-consensual interference is essential, it is equally essential to ensure that strategies are based on evolving empirical findings rather than moral or sociopolitical values [3]. Professional organizations must address this complexity rather than completely disregard it if even a small percentage of people undergo significant change due to SOCE (below morally righteous and willingly involved situations).

This does not imply that SOCE should be approved as a standard or advised intervention. It calls for a more varied and evidence-based approach that distinguishes between client-initiated goals that align with deeply held cultural or spiritual convictions and unethical practices (such as aversive or conversion therapy) [5]. Therefore, Sullins' findings cast doubt on the notion that change-oriented goals are inherently incompatible with psychological well-being [1]. More complex research projects are desperately needed to address this issue, such as mixed-gender, multiethnic, longitudinal, and culturally sensitive studies with validated psychological outcome measures. These studies could clarify which interventions, if any, support adaptive functioning in contexts that are culturally and religiously conservative and involve same-sex attraction.

Conclusion

In summary, despite its limitations, Sullins' research contributes significantly, albeit tentatively, to the ongoing discussion of SOCE. As a result, discussing this subject is not a binary debate but rather a complex problem that engages with belief systems, identity, and therapeutic autonomy. Researchers, policymakers, and mental health professionals must prioritize this topic in order to obtain rigorous, culturally sensitive, and ethically grounded evidence that informs clinical practice and policy development.
